# Establishment of the immunological self in juvenile *Patiria pectinifera* post-metamorphosis

**DOI:** 10.3389/fimmu.2022.1056027

**Published:** 2022-12-06

**Authors:** Mizuki Taguchi, Kota Minakata, Akihiro Tame, Ryohei Furukawa

**Affiliations:** ^1^ Department of Biology, Research and Education Center for Natural Sciences, Keio University, Yokohama, Japan; ^2^ Department of Biosciences and Informatics, Keio University, Yokohama, Japan; ^3^ Department of Marine and Earth Sciences, Marine Works Japan Ltd., Yokosuka, Japan

**Keywords:** coelomocytes, *Patiria pectinifera*, echinoderm, allorecognition, reconstructed chimera, metamorphosis, immunological self

## Abstract

Ontogeny of the immune system is a fundamental immunology issue. One indicator of immune system maturation is the establishment of the immunological self, which describes the ability of the immune system to distinguish allogeneic individuals (allorecognition ability). However, the timing of immune system maturation during invertebrate ontogeny is poorly understood. In the sea star *Patiria pectinifera*, cells that have dissociated from the embryos and larvae are able to reconstruct larvae. This reconstruction phenomenon is possible because of a lack of allorecognition capability in the larval immune system, which facilitates the formation of an allogeneic chimera. In this study, we revealed that the adult immune cells of *P. pectinifera* (coelomocytes) have allorecognition ability. Based on a hypothesis that allorecognition ability is acquired before and after metamorphosis, we conducted detailed morphological observations and survival time analysis of metamorphosis-induced chimeric larvae. The results showed that all allogeneic chimeras died within approximately two weeks to one month of reaching the juvenile stage. In these chimeras, the majority of the epidermal cell layer was lost and the mesenchymal region expanded, but cell death appeared enhanced in the digestive tract. These results indicate that the immunological self of *P. pectinifera* is established post-metamorphosis during the juvenile stage. This is the first study to identify the timing of immune system maturation during echinodermal ontogenesis. As well as establishing *P. pectinifera* as an excellent model for studies on self- and non-self-recognition, this study enhances our understanding of the ontogeny of the immune system in invertebrates.

## Introduction

The role of the immune system is to establish biological individuality and maintain its self-identity. From this perspective, the ontogeny of the immune system is a fundamental issue in immunology. One indicator of immune system maturation is the establishment of the immunological self, which refers to the ability of the immune system to distinguish allogeneic individuals (allorecognition ability). Even in invertebrates, allorecognition ability is well known across a broad range of taxa (1, 2). The best example of invertebrate allorecognition ability is the fusion/non-fusion reaction in colonial animals such as sponges, corals, cnidarians, and ascidians (3–6). These invertebrates possess negligible competence to move away from points of settlement and occasionally live in densely populated communities, leading to frequent encounters with allogeneic individuals (7). When allogeneic contacts begin, the most common outcome is the development of a hemocyte-mediated inflammatory response that destroys vascular continuity between colonies, similar to vertebrate transplant rejection (8). However, in rare cases, individuals may fuse with each other and form a chimeric colony (9). This fusion/non-fusion reaction is determined by the genetic composition of individual colonies (10–12). Similar allorecognition ability has also been reported in solitary animals, such as allograft rejection in the adult sea star *Dermasterias imbricata* (13), cytotoxic reaction in the allogeneic immune cell mixture of the sea urchin *Strongylocentrotus droebachiensis* (14), and a similar “contact reaction” by the allogeneic immune cell mixture of the ascidian *Halocynthia roretzi* (15, 16).

Although there are limited studies on the timing of allorecognition ability acquisition in invertebrates, several studies suggest that the allorecognition system may change ontogenetically (3–6). For example, in the coral *Stylophora pistillata*, adult colonies distinguish accurately between “self” and “non-self” and never fuse with allogeneic colonies (17). However, two types of allogeneic responses have been reported that depend on the age of the interacting partners (17). The first is tissue fusion and the formation of a stable chimera, observed in partners less than two months of age. The second is observed in contacts of 2–4-month-old partners, and leads to fusion and transitory chimera, followed by rejection of chimera partners when the oldest partner in the chimera reaches an age of four months. These results indicate that maturation of allorecognition in *S. pistillata* is achieved through three time-dependent stages, four months after metamorphosis (17). Non-kin fusion between larvae has also been reported in the purple sponge *Haliclona* sp. and *Hydractinia symbiolongicarpus* (18, 19). In *H. symbiolongicarpus*, histoincompatible embryonic chimeras were unstable, with a complete absence of chimerism by four weeks of age following metamorphosis.

A similar tolerance for chimera formation at an early stage of ontogeny has been reported in the sea star *Patiria pectinifera*. Similar to classic studies on individual reconstruction by dissociated sponge cells (20), cells that are dissociated from the embryos or larvae of *P. pectinifera* are capable of reconstructing larvae, similar to sponge cells (21, 22). Allogeneic mixtures of dissociated cells derived from the adult sponge *Callyspongia diffusa* form aggregates but then die within 48 h (23). In contrast, the reconstruction phenomenon in *P. pectinifera* involves a mixture of dissociated cells derived from numerous sibling embryos, indicating that the reconstructed larvae are stable chimeras consisting of siblings. Moreover, we previously revealed that the larval immune system of *P. pectinifera* is tolerant to non-related allogeneic cells (24). This larval immune system comprises a single type of mesenchyme cell that acts as a phagocyte. Mesenchyme cells do not produce an immune response against living allogeneic cells injected into the larval body (24). These findings indicate that the larval immune system recognizes all living allogeneic cells as “self,” regardless of the presence of a kin-relationship.

To date, it remains unclear whether *P. pectinifera* adults display graft rejection between allogenic individuals or graft acceptance between close kin. However, based on the demonstration of allorejection in *D. imbricata* and other invertebrate adults, we hypothesize that *P. pectinifera* adults are also capable of allorecognition. In this study, we demonstrate the allorecognition ability of adult immune cells (coelomocytes) by transplanting allogeneic cells into adult *P. pectinifera*, and clarify the timing of the establishment of an immunological self, which is the basis of allorecognition ability, by inducing metamorphosis in reconstructed chimeras. Here, we report that the immunological self as the basis of allorecognition ability is established at the post-metamorphic juvenile stage, during continued tissue organization to the adult body structure.

## Materials and methods

### Animals

Adult *P. pectinifera* were collected from Tokyo Bay and Mutsu Bay in Japan and kept in a glass aquarium with artificial seawater (ASW; MarineArt SF-1, Tomita Pharmaceutical, Japan) at 15°C. The embryos of *P. pectinifera* were obtained as previously described (25). In brief, mature eggs were prepared by treatment with 1-methyladenine (Sigma-Aldrich, US) (26) and fertilized with diluted sperm. Embryos and larvae were reared in ASW at 20°C with constant rotation at 30 rpm. From two days post-fertilization, the larvae were fed *Chaetoceros calcitrans* (Sun-culture, Marine Tec, Japan) at a concentration of 1.0 × 10^6^ cells/mL and reared to brachiolaria larvae with daily rearing water changes.

Coral sands (approximately 2–5 mm in diameter) from aquarium-reared adult animals were used to induce metamorphosis at the brachiolaria larval stage (27). Metamorphosed larvae were maintained until they reached the juvenile stage.

### Coelomocytes

Coelomic fluid (CF) containing coelomocytes was collected using a needle (26 G × 1/2”: Terumo, Japan) and syringe (medium mouth, 1 mL: Terumo, Japan). The concentration of coelomocytes in the CF used in this study was 3.47 ± 2.06 × 10^5^ cell/mL, as measured by a hemocytometer (C-Chip, NanoEntek, Korea).

To prepare coelomocyte smears, 1 mL of 5 × 10^5^ cells/mL CF containing 0.25 M EDTA were centrifuged at 300 × *g* for 5 min (SC-2, TOMY, Japan) at room temperature (20–25°C). Then, May-Grünwald-Giemsa (MG) staining was performed. The MG method uses May-Grünwald (Merck, US) and Giemsa stain solution (Nacalai Tesque, Japan) containing Azure B and Eosin Y to stain the cell nuclei purple, cytoplasm blue, or pink, and the cytoplasmic granules their specific color. Staining was performed according to the manufacturer’s instructions.

Allogeneic transplantation was performed by injecting 1 mL of coelomocyte-containing CF into five recipients, immediately after collection from a single donor. Autologous transplantation was performed by injecting 1 mL of self-CF (n = 4). In the case of transplanting fluorescently labeled coelomocytes, 1 mL of CF was incubated with 2 µL of 5-(and -6)-carboxyfluorescein diacetate succinimidyl ester (CFSE; 1 mg/mL; DOJINDO, Japan) at 4°C for 45 min. Then, coelomocytes were washed once with ASW by centrifugation at 1,000 × *g* for 5 min at 4°C, and resuspended in 1 mL of sterilized ASW.

To observe the immune response after coelomocyte transplantation, 200 µL of CF was collected in a Petri dish with 2 mL of sterilized ASW and allowed to stand for 30 min at 20°C. When fixing the coelomocytes and their aggregate form, the collected CF was immediately mixed with an equal volume of 4% w/w paraformaldehyde-containing ASW (PFA-ASW) for bright-field or confocal microscopy or 5% w/w glutaraldehyde-containing ASW for electron microscopy.

Based on the microscopic images, the area and number of coelomocyte aggregates were measured using ImageJ software (version:1.53j). Aggregates were defined as having an area of more than 2,000 µm^2^, which corresponds to an area of approximately 10 coelomocytes of the major cell type arranged on a flat surface.

### Preparation of chimeric larvae

Blastulae (approximately 16 h post-fertilization) obtained from the two parent pairs were fluorescently labeled with chloromethylfluorescein-diacetate (CMFDA, 100 µM; AAT Bioquest, US) or carboxyfluorescein diacetate (CFDA, 100 µM; AAT Bioquest, US) for 30 min at 20 °C. These embryos, having a packed volume of 0.2 mL, were dissociated by a previously described method (21), then reconstructed independently or in a mixture. The definition of the chimeras produced in this study is shown in **Supplementary Figure 1**. The reconstruction was performed in a glass Petri dish at 15°C. After specimens formed a mouth, they were fed with *C. calcitrans* (Section 2.1), until they reached the brachiolaria stage. The fluorescently labeled specimens were observed with a laser confocal microscope (Fluoview, Olympus, Japan). For observations under the electron microscope, juveniles that transformed from the larvae stage were fixed with 2.5% glutaraldehyde in ASW.

### Electron microscopy

Coelomocyte aggregates and juveniles fixed with glutaraldehyde were embedded in 1.0% agarose and post-fixed with 2.0% osmium tetroxide in filtered ASW for 2 h at 4°C. After washing with distilled water, the sections were dehydrated using a graded ethanol series and embedded in Epon 812 (TAAB) at 65°C. Ultra-thin sections (70 nm thickness) of coelomocytes and coelomocyte aggregates were cut using a diamond knife on an Ultracut S ultra-microtome (Leica, Germany), stained with 2.0% uranyl acetate solution and 2.0% lead citrate solution, and observed using a transmission electron microscope (Tecnai G2 20, FEI, US) operating at 200 kV. For the juveniles, semi-thin sections (thickness, 500 nm) of juveniles were cut using a diamond knife, stained with 0.1% toluidine blue in 0.1 M phosphate buffer [pH 7.4], and observed using a BX-51 light microscope (Olympus, Japan). Juvenile sections were stained with uranyl acetate and lead citrate solutions, coated with osmium using an OPC80 osmium coater (Filgen, Japan), and observed using a field-emission scanning electron microscope (Quanta 450 FEG; FEI, US) with a backscattered electron detector operating at 5 kV.

### Statistical analyses

Survival time was measured after metamorphosis of the reconstructed chimeras. Death was determined by morphological distortion and atrophy in addition to motility, including tube foot movements. Kaplan–Meier survival curves were used to visualize the data, and a log-rank test was performed with *p* < 0.05 considered statistically significant. R (version 4.2.0) software was used for all statistical analyses (28).

## Results

### Characterization of coelomocytes in *P. pectinifera*

Coelomocytes are the cells present in the CF of echinoderms that mediate immune responses. The various types of coelomocyte are characterized by their morphology, and include phagocytes, spherule cells (also called spherulocytes, morula cells, and granulocytes), and progenitor cells (29–31). However, the coelomocytes in *P. pectinifera* remain uncharacterized.

**Figure 1:**
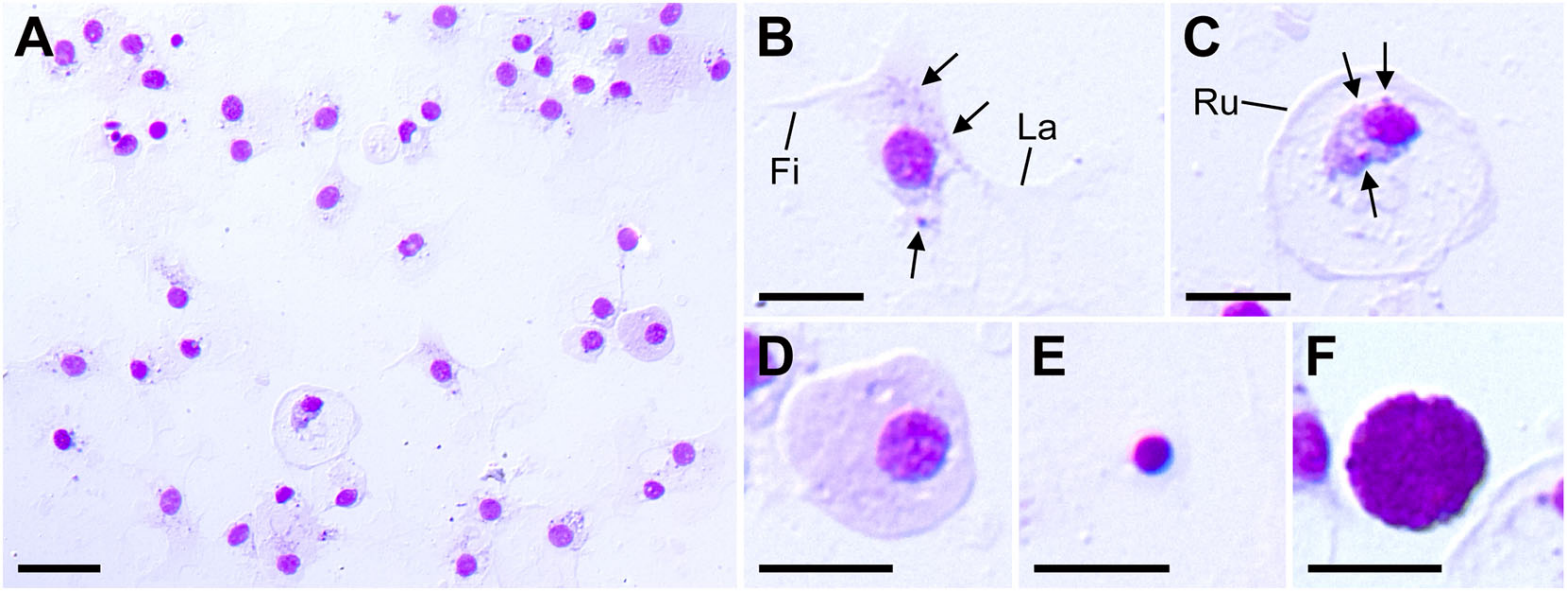
*Patiria pectinifera* coelomocytes. **(A)** Coelomocytes smear stained by MG. **(B–F)** Five identified cell types: **(B)** amoebocyte; **(C)** discoidal phagocyte; **(D)** agranulocyte; **(E)** progenitor cell; **(F)** morula cell. Fi, filopodia; La, lamellipodia; Ru: membrane ruffling, Arrows: granules. Scale bars: 20 µm **(A)**, 10 µm **(B–F)**.

Here, we attempted to classify coelomocytes based on morphology and MG staining (**Figure 1A**). At least five types of coelomocytes were discriminated using MG staining. The first type were cells that comprised the majority of the smear and appeared in contact with each other in a network-like manner (**Figures 1A, B**). These cells contained small granules and adhered to the substrate *via* widely spreading lamellipodia, with filopodia observed in some cells (**Figure 1B**). These features suggested that these cell types were the phagocytes corresponding to amoebocytes (also called petaloid phagocytes), which are most abundant in the CF of other echinoderms (31–33). The second and third cell types both presented a disc-shaped morphology similar to the discoidal phagocytes in sea urchin (34); however, differences in the stainability were clearly observed (**Figures 1C, D**). In the cells depicted in **Figure 1C**, the nucleus size was relatively small compared to the cells in **Figures 1B, D**, indicating condensed chromatin. These cells included small granules, and the cytoplasm stained light pink was localized near the nucleus. Membrane ruffling was observed at the periphery of the lamellipodia, indicating that these cells adhered to the substrate with their membranes spread to the full extent. In contrast, the third cell type had a large nucleus and evenly stained cytoplasm, with no observed granules or membrane ruffling (**Figure 1D**). Therefore, we concluded that the second cell type was discoidal phagocyte and the third cell type was agranulocytes. The fourth cell type shown in **Figure 1E** was small with a high nuclear-cytoplasmic ratio, which is characteristic of undifferentiated cells, corresponding to progenitor cells in other echinoderms (29–31). The fifth cell type was spherical, and the cytoplasm was filled with many basophilic granules (**Figure 1F**), consistent with the characteristics of morula cells in other echinoderms (31, 35). Although no nucleus was visible in **Figure 1F**, the presence of the nucleus was confirmed in the other cells (**Supplementary Figure 2**). This cell type was present in only approximately 0.02% of the smear.

### Coelomocytes form aggregates in response to injected allogeneic coelomocytes and phagocytose the allogeneic cells

**Figure 2:**
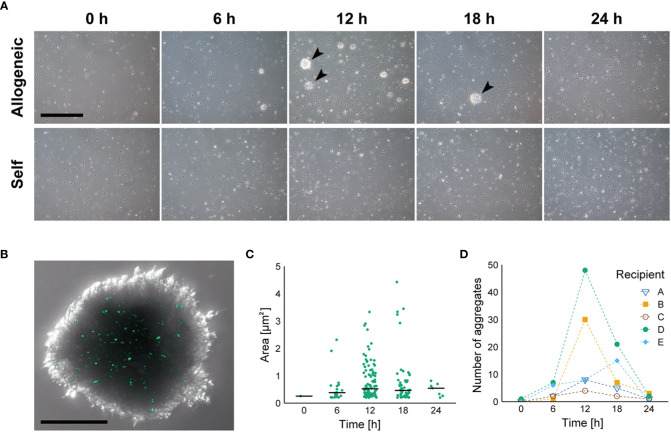
Dynamics of coelomocyte aggregate formation in response to injected allogeneic coelomocytes. **(A)** Time course of allorecognition response to allogeneic coelomocytes. Arrow heads indicate coelomocyte aggregates. Scale bar: 500 µm. **(B)** Magnified image of coelomocyte aggregate in response to fluorescently labeled allogeneic coelomocytes. Scale bar: 100 µm. **(C)** Temporal change in the size of coelomocyte aggregates, shown in **(A)**, after injection of allogeneic coelomocytes. Horizontal bars indicate the median size at each time interval. **(D)** Temporal change in the number of coelomocyte aggregates after injection of allogeneic coelomocytes in each recipient.

To verify the allorecognition ability of coelomocytes, we injected fluorescently labeled allogeneic coelomocytes into the coelom of recipient animals. Aggregates of coelomocytes developed over time in the recipient’s CF, and fluorescent signals derived from the injected cells were observed in the aggregates with a scattered pattern (**Figures 2A, B**). In contrast, in the case of injected self-coelomocytes, no aggregate was observed (**Figure 2A**). These results clearly indicated that the coelomocytes of *P. pectinifera* possessed allorecognition ability. The size and number of aggregates peaked at 12 h after the allograft then decreased (**Figures 2C, D**). The number of aggregates varied widely among individuals (**Figure 2D**); however, the aggregates from individuals with a small number of aggregates tended to be large, and a large variation was observed in aggregate size, especially at 12 h and 18 h.

**Figure 3:**
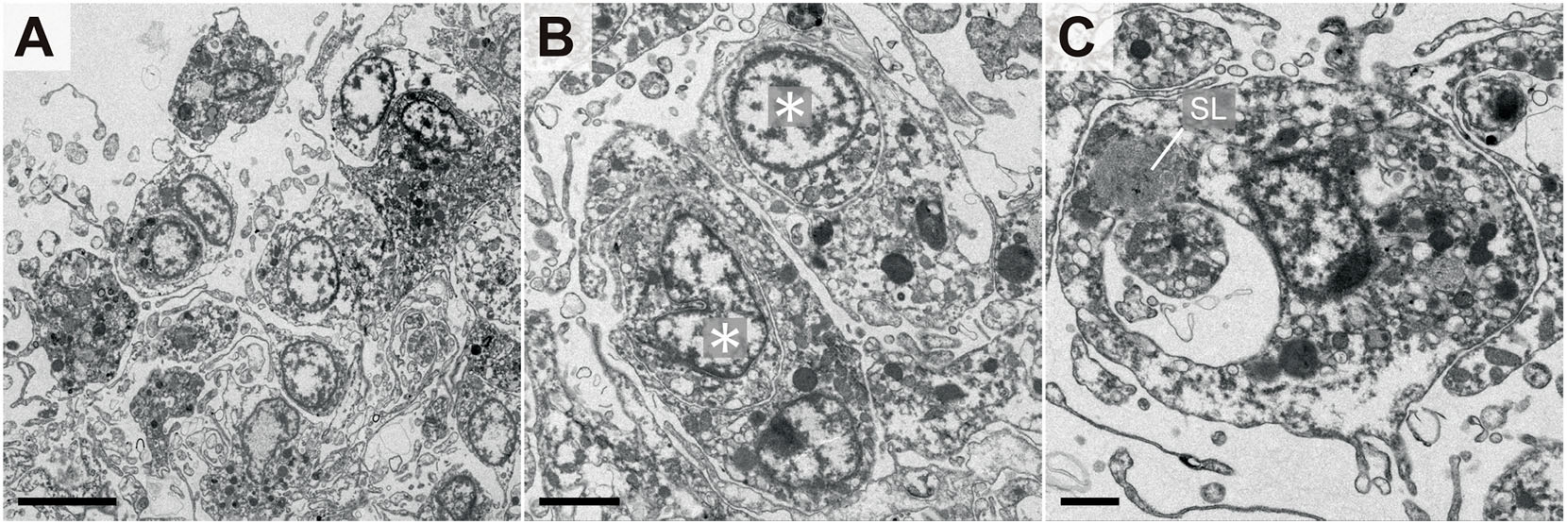
Internal morphology of coelomocyte aggregates in response to allogeneic coelomocytes. **(A)** Outer edge of aggregate. **(B)** Coelomocytes that phagocytosed other coelomocytes. Asterisks indicate the nuclei of phagocytosed coelomocytes. **(C)** Coelomocyte during digestion of the phagocytosed cell. A secondary lysosome (SL) that fused with the endosome was also observed. Scale bars: 5 µm **(A)**, 2 µm **(B)**, 1 µm **(C)**.

**Figure 3** demonstrates the internal morphology of the aggregates. Each coelomocyte in the aggregate was densely packed, but no multinucleated cells were observed, whereas numerous cross-sections of the pseudopodia were observed in the intercellular spaces (**Figure 3A**). Coelomocytes that independently phagocytosed other cells were scattered inside the aggregates (**Figure 3B**). Moreover, coelomocytes containing secondary lysosomes were observed (**Figure 3C**). These results corresponded to the detected pattern of fluorescent signals derived from allografted cells, as indicated in **Figure 2B**. To determine whether such aggregate formation without cell fusion occurred only in response to allogeneic heterologous cells, the bacteria were injected into the adults. As a result, coelomocytes were shown to aggregate without cell fusion, and each coelomocyte phagocytosed the bacteria individually (**Supplementary Figure 3**).

### Chimeric larvae develop into juveniles through a normal metamorphosis process

The normal individual reconstruction processes performed by the dissociated cells of *P. pectinifera* embryos are as follows: (i) the mixture of dissociated cells reaggregates within 2 h after dissociation; (ii) 8 h after the start of reconstruction, a germ layer forms *via* the sorting of ectodermal and endodermal cells; and (iii) by 24–40 h, as the blastocoel gradually expands, the ectoderm invaginates and eventually fuses with the endoderm, forming a blastopore. After these reconstruction processes, the reconstructed embryos develop normally into bipinnaria larvae, forming mouth and coelomic pouches (21). This reconstruction phenomenon is also achieved by mixing dissociated cells from sibling embryos derived from a common parent (22).

**Figure 4:**
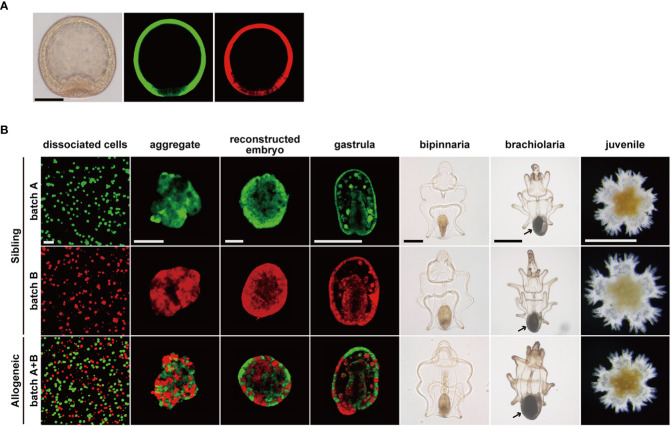
Developmental process of chimeric larvae. **(A)** Fluorescent labeled blastulae before dissociation. Images of differential interference contrast (left), stained by CMFDA (middle), stained by CFDA (right). Scale bar: 100 µm. **(B)** Developmental processes of reconstructed chimeras. Arrows: adult rudiment. Scale bars: 40 µm (dissociated cells to reconstructed embryo), 200 µm (gastrula and bipinnaria), 500 µm (brachiolaria and juvenile).

In this study, we first stained blastulae derived from two pairs of parents (batches A and B) using different fluorescent dyes (**Figure 4A**; **Supplementary Figure 1A**). The sibling embryos were dissociated and reconstructed individually (**Figure 4B**, batch A or B) or mixed (**Figure 4B**, batch A + B). The reconstructed embryos from batches A and B were sibling chimeras and developed normally into bipinnaria larvae, as previously reported (21). In contrast, although the reconstructed embryos from a cell mixture of batches A and B were chimeras comprising cell populations without kin-relationships, these allogeneic chimeras also successfully developed into bipinnaria larvae, as expected from our previous research (24), in which the larval immune system was tolerant of living allogeneic cells (**Figure 4B**, allogeneic chimera). We observed no difference in morphogenesis between the sibling chimeras (**Figure 4B**). However, in the reconstructed embryos of allogeneic chimeras, we noted that the fluorescent signals derived from each batch were distributed in a patchy fashion, indicating that cells derived from each batch tended to accumulate separately.

Upon feeding, bipinnaria larvae developed into brachiolaria larvae able to metamorphose, and adult rudiment developed at the posterior end of the larvae (**Figure 4B**, arrows). Regardless of the presence or absence of a kin-relationship, all chimeras developed into brachiolaria larvae. When we induced these chimeras to metamorphose, all chimeras, including allogeneic chimeras, reached the juvenile stage within two days through the typical metamorphic process of *P. pectinifera* (**Figure 4B**, juvenile). No abnormalities were found in the external morphology of the chimeric juveniles. Moreover, we observed no statistically significant difference in body length between the sibling and allogenic chimeras throughout the developmental stages (**Supplementary Figure 4**).

### Allogeneic chimeras die one month after metamorphosis to the juvenile stage

**Figure 5:**
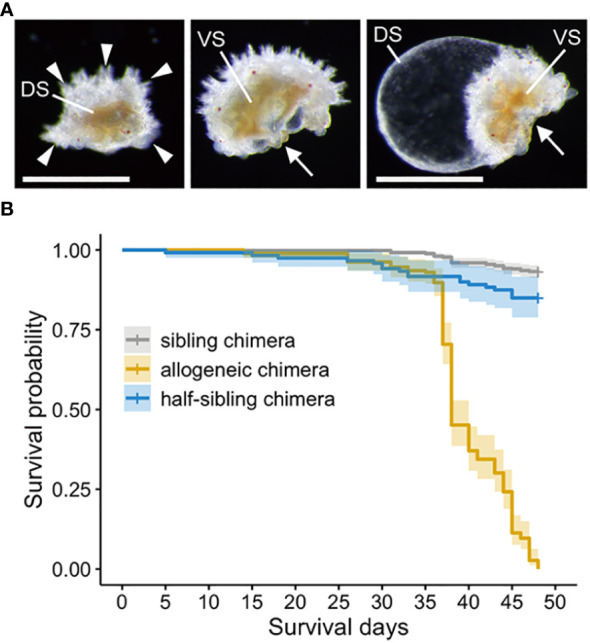
Abnormal morphologies and survival time analysis of allogeneic chimera juveniles. **(A)** Allogeneic chimera showing abnormal morphologies. Left: view of back bridge form from above. Middle: bent in half. Right: swelling like balloons. DS, dorsal side; VS, ventral side; Arrowheads: five arms. Arrows: mouth. Scale bar: 500 µm. **(B)** Survival time curve of the juvenile stage using the Kaplan–Meier method.

Individuals in the allogeneic chimeras with abnormal morphologies, such as those bent in half or some swollen balloons, gradually emerged two weeks after reaching the juvenile stage. (**Figure 5A**). These morphologies were not observed in sibling chimeras. Furthermore, one month after metamorphosis, the number of dead individuals increased rapidly in allogeneic chimeric juveniles only, with all allogeneic chimeras eventually dying. Many of the juveniles that died during this period did not show any morphological abnormalities, as mentioned above; however, the motility of their tube feet and their adhesive property to coral sands, which is a metamorphosis-induced substrate, were completely lost. **Figure 5B** depicts the survival time curve of the juvenile stage using the Kaplan–Meier method. Using a log-rank test, we observed a significant difference in the survival time between sibling and allogeneic chimeras, and the hazard risk of allogeneic chimeras was 40.901 times (95% CI: 28.654–58.38) higher than that of sibling chimeras. However, in a preliminary experiment conducted in other batches, allogeneic chimeric juveniles were completely destroyed in approximately two weeks (**Supplementary Table 1**). A batch effect may exist for the time to death of the allogeneic chimeric juveniles.

To investigate whether the death of allogeneic chimeras was caused by genetic incompatibility, we prepared half-sibling chimeras with a different mother or father (**Supplementary Figure 1B**). These half-sibling chimeras also developed normally into brachiolaria larvae and showed no morphological abnormalities after metamorphosis to the juvenile stage, as with the allogeneic chimeras. Although we observed a significant difference in the mortality in the juvenile stage of half-sibling chimeras compared to that of the sibling chimeras (hazard risk: 2.348 fold, 95% CI: 1.354–4.07), and the survival rate was much higher than that of the allogeneic chimeras (hazard risk of allogeneic chimeras to half-sibling chimeras: 22.27 fold, 95% CI: 13.16–37.69) (**Figure 5B**).

### Allogeneic chimeric juveniles exhibit loss of the epidermal cell layer and increased cell death in the digestive tract

**Figure 6:**
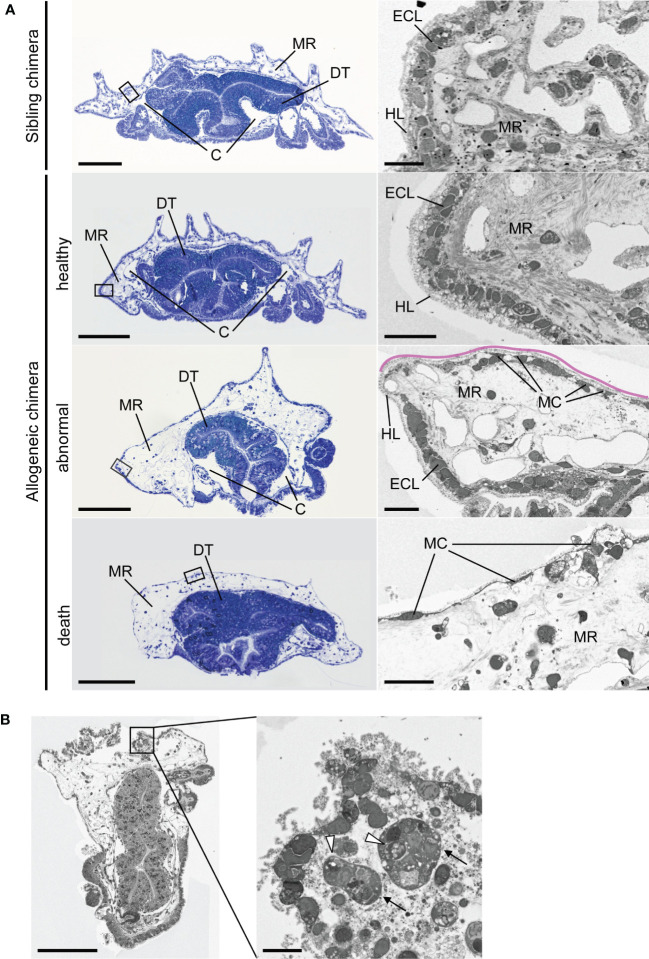
Internal morphology of chimeric juveniles. **(A)** Light microscopic and transmission electron microscopic images of semi-thin sections of juveniles. Left column: whole juvenile sections stained by toluidine blue. Sibling chimeras and healthy individual are 5 dpm, abnormal individual is 14 dpm, and death occurred at 30 dpm. Ventral side is downwards. Enlarged portion of the black box is shown in the right column. Pink lines indicate that part of the epidermal cell layer was lost. C, coelomic cavity; DT, digestive tract; ECL, epidermal cell layer; HL, hyalin layer; MC, mesenchyme cell; MR, mesenchymal region. **(B)** Phagocytosis in areas of severe damage in epidermal cell layer of abnormal-morphology individual. Arrows: cells containing phagosome-like vesicles. Arrowheads: vesicles. Scale bars: 100 µm (left column in **A** and **B**), 10 µm (right column in **A** and **B**).

We examined the internal morphology of allogeneic chimera juveniles in the process of death. An epidermal cell layer was present near the body surface of the healthy sibling chimera juvenile five days post-metamorphosis (dpm); beneath this cell layer, we observed condensed mesenchymal regions corresponding to larval blastocoel filled with extracellular matrix (**Figure 6A**). At this time, skeletal plate formation, which is a characteristic of echinoderms, had not yet been observed, and a hyaline layer derived from the larvae covered the dorsal surface of the individual. Furthermore, the vast coelomic cavity, a major characteristic of the adults, was still underdeveloped. These morphologies revealed that the post-metamorphic juvenile continued tissue organization for a period of time. Similar morphologies were also observed in the allogeneic chimera five days after development into the juvenile (**Figure 6A**), with no difference in morphological characteristics between sibling chimeras and allogeneic chimeras within five days after metamorphosis.

In contrast, in the allogeneic chimera juvenile, the mesenchymal region of the individuals with morphological abnormalities (**Figure 5A**) indicated abnormal expansion and was not condensed, as shown in the sibling juvenile (14 dpm) (**Figure 6A**; abnormal, MR). Furthermore, part of the epidermal cell layer was lost, and only a hyalin layer-like structure was observed (**Figure 6A**, pink line). In this region, larval mesenchyme cells, which developed pseudopodia, were observed along the hyalin layer (**Figure 6A**, MC). This observation was not limited to allogeneic chimeras with morphological abnormalities; the mesenchymal region was also expanded in chimeric individuals that died without showing deformation (30 dpm) (**Figure 6A**, death). Conversely, development of the coelomic cavity was not observed. Furthermore, most of the epidermal cell layers of dead allogeneic chimera juveniles, with or without morphological abnormalities, disintegrated, and only larval mesenchyme cells remained along the hyalin layer (30 dpm) (**Figure 6A**; death, MC). In areas of severe tissue damage, we observed many collapsed cells and vesicles that appeared to have originated from these cells, as well as cells containing phagosome-like vesicles (**Figure 6B**).

**Figure 7:**
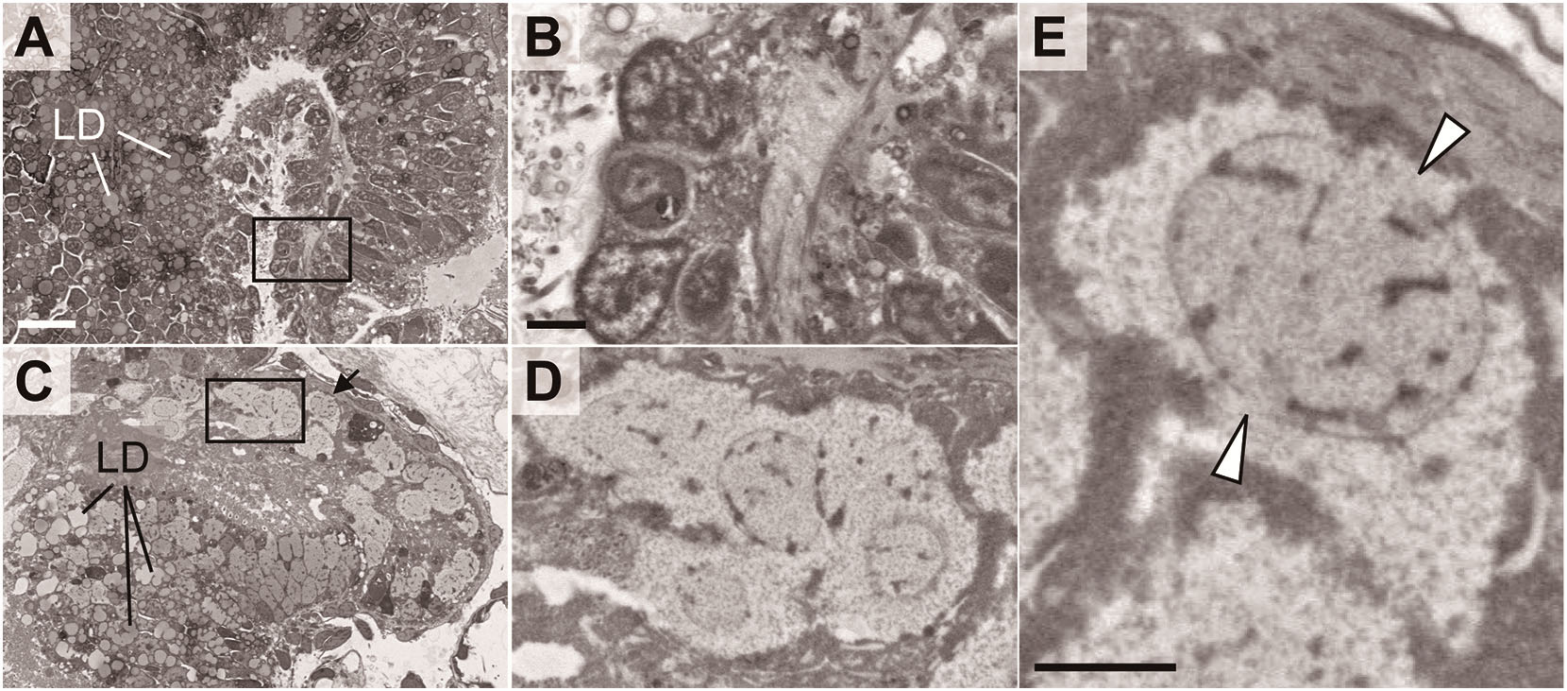
Abnormal morphologies of digestive tract in chimeric juveniles. Sibling chimera **(A, B)** and dead allogeneic chimera **(C–E)** shown in **Figure 6**. LD, lipid droplet. Black boxes in **A, C** are shown enlarged in **B, D**, respectively. **(E)** Cell indicated by arrow in **(C)** Arrowheads: segmented nuclear membrane. Scale bars: 10 µm **(A)**, 2 µm **(B, E)**.

**Figure 7** indicates a magnified electron microscopic image of the digestive tract of the juveniles. In the sibling chimeras, the intestinal cells contained nuclei that exhibited a relatively high electron density and numerous lipid droplets (**Figures 7A, B**). In contrast, such cells were not observed in allogeneic chimeras, whereas numerous cells contained enlarged nuclei and cytoplasm (**Figure 7C, D**). Moreover, in some cells, we observed uniform chromatin with low electron density, nuclear membrane damage, an absence of distinct cell membrane, and loss of organelles, suggesting that these cells were dead (**Figures 7D, E**).

## Discussion

In a previous study, we reported that the larval immune cells of *P. pectinifera*, i.e., the mesenchyme cells, do not exhibit an immune response to live allogeneic cells (24). In this study, we clearly demonstrate that the adult coelomocytes phagocytose injected allogeneic cells with aggregate formation, suggesting that the adult immune system recognizes allogeneic cells as non-self. As injected donor coelomocytes possess allorecognition capabilities, the recipient’s cells should be also phagocytosed. However, no host individuals were severely affected by the transplantation. The results of transplanting fluorescently labeled cells suggest that, even if some donor cells phagocytose the host cells, they are eventually incorporated into the aggregate, thereby minimizing the effect on the host (**Figure 2B**).

In *P. pectinifera*, the peak time for aggregate formation of coelomocytes in response to injected allogeneic cells was 12–18 h after transplantation (**Figure 2C**), which is approximately consistent with Bertheussen’s observation of allogeneic cytotoxicity in *Strongylocentrotus droebachiensis* coelomocytes from approximately 20 h after mixing with allogeneic coelomocytes *in vitro* (14). As the cytotoxic activities of *P. pectinifera* coelomocytes were not verified in this study, further analysis of CF during the allorecognition response is required. However, individual differences in the size of aggregates were observed during the allorecognition response in *P. pectinifera* coelomocytes, despite the transplantation of allogeneic cells derived from the same donor. Although it is possible that this result reflects genetic distance to the donor, it should be noted that we used wild animals maintained in a non-sterile environment, and the cell density in the CF at the start of the experiment was initially different (see also section 2.2). Nevertheless, the detailed dynamics of the allorecognition response should be further verified *in vitro*.

Coelomocytes are the only cells of the coelomic cavity in echinoderms and serve as immune cells in adults. Coelomocytes have been classified into six major groups: phagocytes, spherule cells, hemocytes, progenitor cells, crystal cells, and fusiform cells (36). Amoebocytes and discoidal phagocytes, which are classified in this study, are phagocytes reported in almost all echinoderms (31, 37, 38). Amoebocytes, in particular, are the major coelomocytes of echinoderms. Both are reportedly involved in clotting, encapsulation, chemotaxis, opsonization, and graft rejection, in addition to phagocytosis (36). According to the internal structure of the aggregates observed in this study, the intercellular spaces were filled with structures that appear to be cross-sections of pseudopodia, suggesting that most of the coelomocytes participating in the aggregate formation are amoebocytes. Although no multinucleated cells were observed, Dan et al. reported that echinoderm phagocytes attached to the substrate surface show a high fusogenic capability (39). This phenomenon represents the encapsulation of large foreign bodies that cannot be phagocytosed by individual cells. We suggest that that the reason the fusion of amoebocytes was not observed in this study was because we employed foreign bodies of a size that could be phagocytosed by individual cells. In addition, cells corresponding to the agranulocytes classified in this study have not previously been reported in echinoderms. These agranulocytes are clearly distinct from discoidal phagocytes in terms of the differences in the nucleus size and cytoplasmic stainability, in addition to an absence of granules. Considering their ability to spread their membranes into disks, the agranulocytes may also have phagocytic ability. The fourth cell type, progenitor cells, termed lymphocytes in some echinoderms, have also been reported in most echinoderms (31, 38). In this study, we also observed morula cells in very low proportions in the smears. These cells were filled with basophilic granules and may correspond to the type I granulocyte recently described in the sea cucumber *Apostichopus japonicus* (31). Morphologically, the morula cells are very similar to spherule cells in *Crossaster granularis* (35). In sea urchins, although red spherule cells exhibit antibacterial activity (40), the functions of colorless spherule cells remain unknown. To elucidate their function, further analysis of the constituents in the granules of *P. pectinifera* morula cells is required. Other major coelomocytes in echinoderms, hemocytes, crystal cells, and fusiform cells were not observed in the smears in this study. These cells were likely to be relatively small floating cells in the CF that could not be observed using the employed smear preparation method.

The transition from a larval immune system that is tolerant of allogeneic cells to an adult immune system with allorecognition capabilities implies that the immunological self is established before and after metamorphosis. In the three chimeric larvae with different allogeneic levels, sibling and half-sibling chimeras showed high survival rates, whereas all allogeneic chimeras died (**Figure 5**). The difference in allogeneic levels between these chimeras was based on the number of shared parental haplotypes; sibling chimeras from the same parents included four haplotypes, half-sibling chimeras with one shared parent included six haplotypes, and allogeneic chimeras from two sets of parents included eight haplotypes. If we assume that chimeras are tolerated if at least one of the haplotypes of the histocompatibility genes (assuming a gene complex) is compatible in *P. pectinifera*, as predicted from the homologous recognition responses of colonial animals such as sponges and ascidians (41–45), then each cell comprising sibling, half-sibling, and allogeneic chimeras will share at least one haplotype with 75.0%, 67.5%, and 37.5% of cells in the same individual, respectively. Consequently, the probability of neighboring cells sharing a haplotype is high for sibling and half-sibling chimeras, but low for allogeneic chimeras. This difference in the probability of histocompatibility may explain the difference in survival rates between sibling/half-sibling chimeras and allogeneic chimeras.

Our observations of the internal morphology of the chimeric juveniles indicated that tissue organization, such as expansion of the coelomic cavity with narrowing of the mesenchymal region derived from the larval blastocoel, continues for a period of time during the post-metamorphic juvenile stage (**Figure 6**). During this process, extremely characteristic morphological changes were observed in the bodies of deceased allogeneic chimeras (**Figure 6A**, allogeneic chimera). The most significant feature was that some of the epidermal cell layers were missing, and the mesenchymal region was abnormally distended. At present, it is unclear whether these morphological changes seen in the allogeneic chimeras are caused by mutual attack by coelomocytes derived from the different parents, or tissue dysfunction owing to histocompatibility differences. In the sponge *C. diffusa*, apposition of cell aggregates derived from separate individuals within 8 h after initiation of aggregation by dissociated cells results in cytotoxic mutual destruction (23). In *P. pectinifera*, phagocytosis was also observed around the severely damaged epidermal tissue, suggesting activation of the immune system; however, no indication of an immune response in the digestive tract was evident, wherein tissue incompatibility appeared to cause non-apoptotic regulatory necrosis (46). In the hydroid *Hydractinia symbionlogicarpus*, the allorecognition response triggers autophagy and necrosis (47). Regulatory necrosis caused by tissue incompatibility may also occur first in *P. pectinifera*, thereby activating the immune response. In any case, this response may not occur without establishment of the immunological self. As the allogeneic chimeras died while tissue organization was in progress, we suggest that the immunological self of the sea star is established prior to the completion of adult tissue organization. Moreover, we speculate that the adult-type (coelomocyte) immune system may be involved in this organization.

Importantly, cell populations derived from each parent aggregated separately and were patchily distributed in the reconstructed embryos of allogeneic chimeras. That is, sorting by genotype occurred within a single individual rather than into multiple individuals. This suggests not only that differences in histocompatibility already exist in the embryonic cells, but also that both populations can function cooperatively up to the juvenile stage, regardless of tissue compatibility or incompatibility. Allorecognition in *Botryllus schlosseri* involves a gene complex called fuhc, which contains genes with polymorphisms that co-segregate with allorecognition responses, as well as genes that do not co-segregate with allorecognition responses (8, 12, 48–50). It is possible that multiple genes were also involved in histocompatibility in *P. pectinifera*, with only some of these genes expressed during the embryonic and larval stages. Subsequently, all histocompatibility genes were expressed in the post-metamorphic juvenile stage of *P. pectinifera*, and the allorecognition system began to operate, which likely formed the basis of the establishment of the immunological self.

Chimerism of allogeneic larvae has also been described for another invertebrate, the hydroid (19). In the case of *Hydractinia*, as in the present study, histoincompatible chimeras exhibited reduced survival. Moreover, the chimerism of histoincompatible chimeras was gradually lost, and even completely lost in some surviving individuals. In contrast, a similar loss of chimerism did not occur in the allogeneic chimeras of *P. pectinifera* because all individuals died from the likely large number of haplotypes in the individuals. Conversely, it may be possible to control the survival rate of the allogeneic chimeras by varying the mixing ratio of allogeneic cells for reconstruction, i.e., the ratio of the haplotypes present. If possible, this may provide a useful method of identifying genomic differences between individuals.

In conclusion, we demonstrated that the *P. pectinifera* immune system matures during the post-metamorphic juvenile period and switches from a larval immune system that recognizes cells of the same species as the self to an immune system centered on coelomocytes that have allorecognition ability. Because the reconstructed chimeras produced in this study were based on dissociated cells derived from multiple embryos, we could not fully confirm the establishment of an immunological self. However, by further developing a reconstructive system utilizing a single individual, rigorous allogenic chimeras may be created, which would enable the identification of self-markers *via* a genomic approach. Additionally, further studies are required to determine the tissues from which the coelomocytes are differentiated in juveniles, which form the basis of the adult immune system. Future research will focus on changes in the transcriptome during metamorphosis, which will reveal the molecular mechanisms that trigger development of the adult-type immune system. Our study provides a valuable model for studying not only the ontogeny of the immune system, but also the mechanisms underlying self- and non-self-recognition in invertebrates.

## Data availability statement

The original contributions presented in the study are included in the article/**Supplementary Material**. Further inquiries can be directed to the corresponding authors.

## Author contributions

MT, KM, and RF designed the experiments. MT and KM were involved in coelomocyte classification. KM conducted graft experiments supervised by MT and RF. MT performed the reconstruction experiments on dissociated embryonic cells. AT conducted the histochemical analysis and electron microscopy. All the authors contributed to the analysis of the experimental results and manuscript writing. The entire project was coordinated by MT and RF. All authors contributed to the article and approved the submitted version.

## Supplementary material

Supplementary Figure 1 Definition and preparation scheme of chimeric larvae. **(A)** Preparation of sibling and allogeneic chimeras. **(B)** Preparation of half-sibling chimeras with a different mother or father. Numbers indicate individuals.

Supplementary Figure 2 Morula cell with visible nucleus in coelomocyte smears. Nucleus stained reddish purple by MG staining was identified in the cytoplasm filled with basophilic granules. N: nucleus. Scale bar: 10 µm.

Supplementary Figure 3 Coelomocyte that formed an aggregate in response to injected *Escherichia coli*. Magnified image of outer edge of the aggregate. Bacterial challenge was performed according to a previously described method [Furukawa et al., *Dev Comp Immunol.* (2012) 36, 51–61]. Arrowheads indicate phagocytosed bacteria. Similar to the allorecognition response, the interspace of each coelomocyte that phagocytosed the bacteria was filled with pseudopodia, indicating that the coelomocytes did not fuse with each other but phagocytosed the bacteria individually. Scale bar: 2 µm.

Supplementary Figure 4 Body length of chimeric individuals in each developmental stage. Body length (long diameter) of reconstructed chimera was measured and plotted. No significant differences were detected in each developmental stage according to analysis of variance (ANOVA) tests.
