# Supplementary figures and images for: Establishment of the immunological self in juvenile Patiria pectinifera post-metamorphosis
Source: Front Immunol. 2022 Dec 6;13:1056027. doi: 10.3389/fimmu.2022.1056027 (PMC9763293; doi:10.3389/fimmu.2022.1056027)

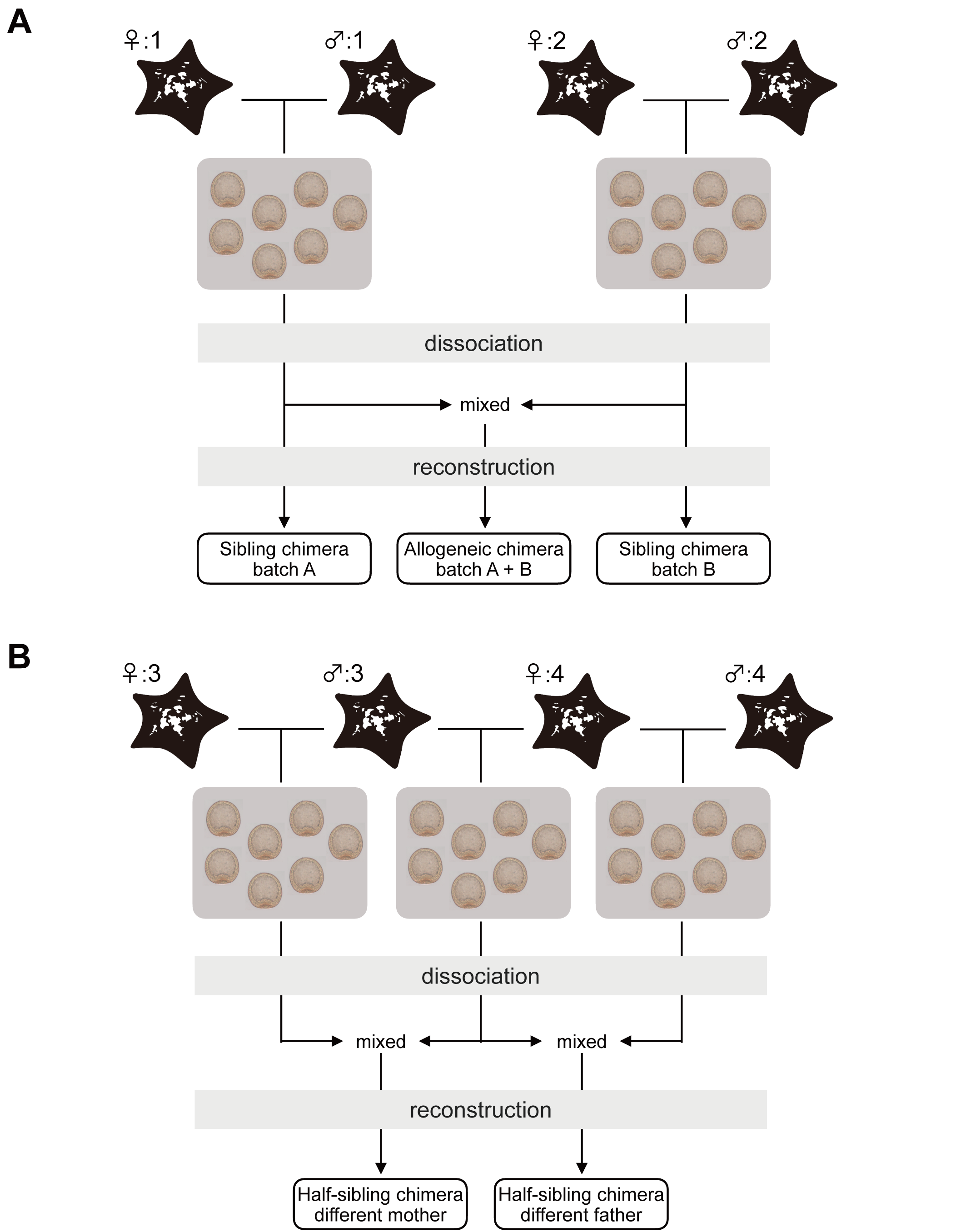

Supplement: Supplementary Figure 1 — Definition and preparation scheme of chimeric larvae. (A) Preparation of sibling and allogeneic chimeras. (B) Preparation of half-sibling chimeras with a different mother or father. Numbers indicate individuals. [file Image_1.tif]

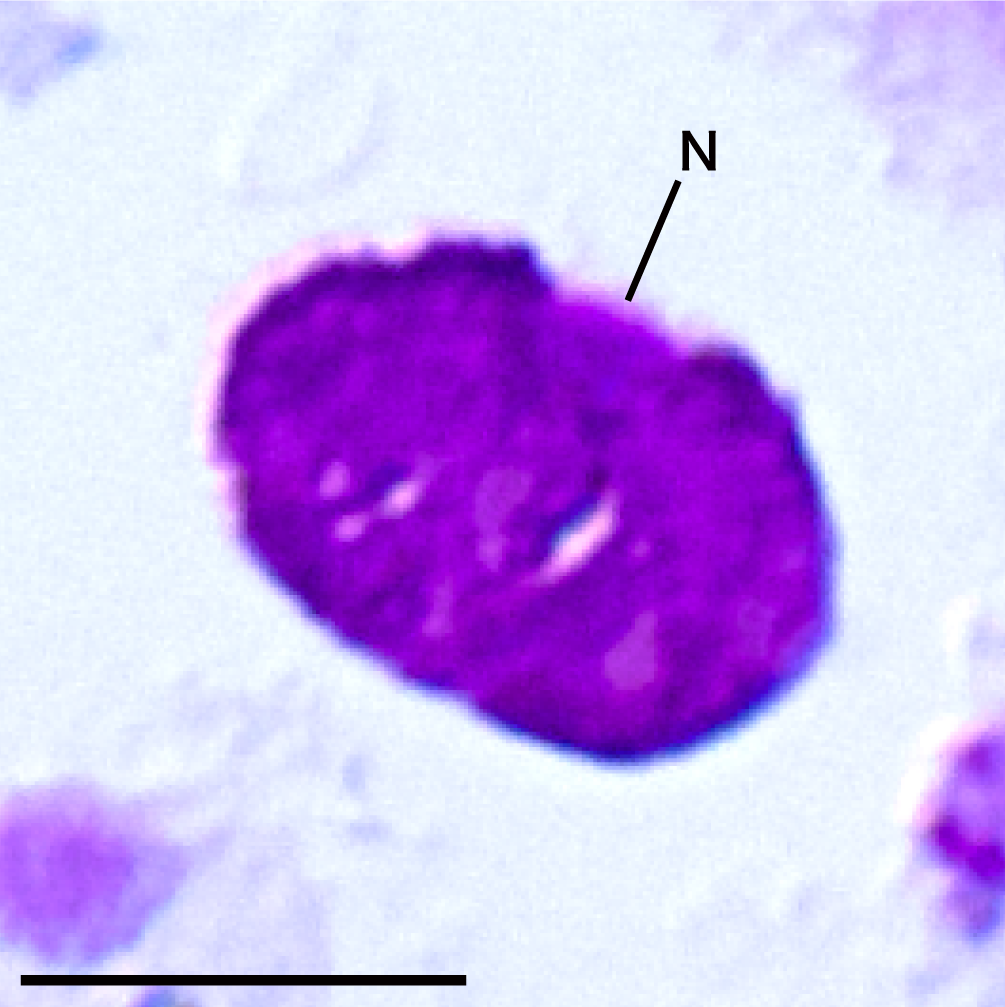

Supplement: Supplementary Figure 2 — Morula cell with visible nucleus in coelomocyte smears. Nucleus stained reddish purple by MG staining was identified in the cytoplasm filled with basophilic granules. N: nucleus. Scale bar: 10 µm. [file Image_2.tif]

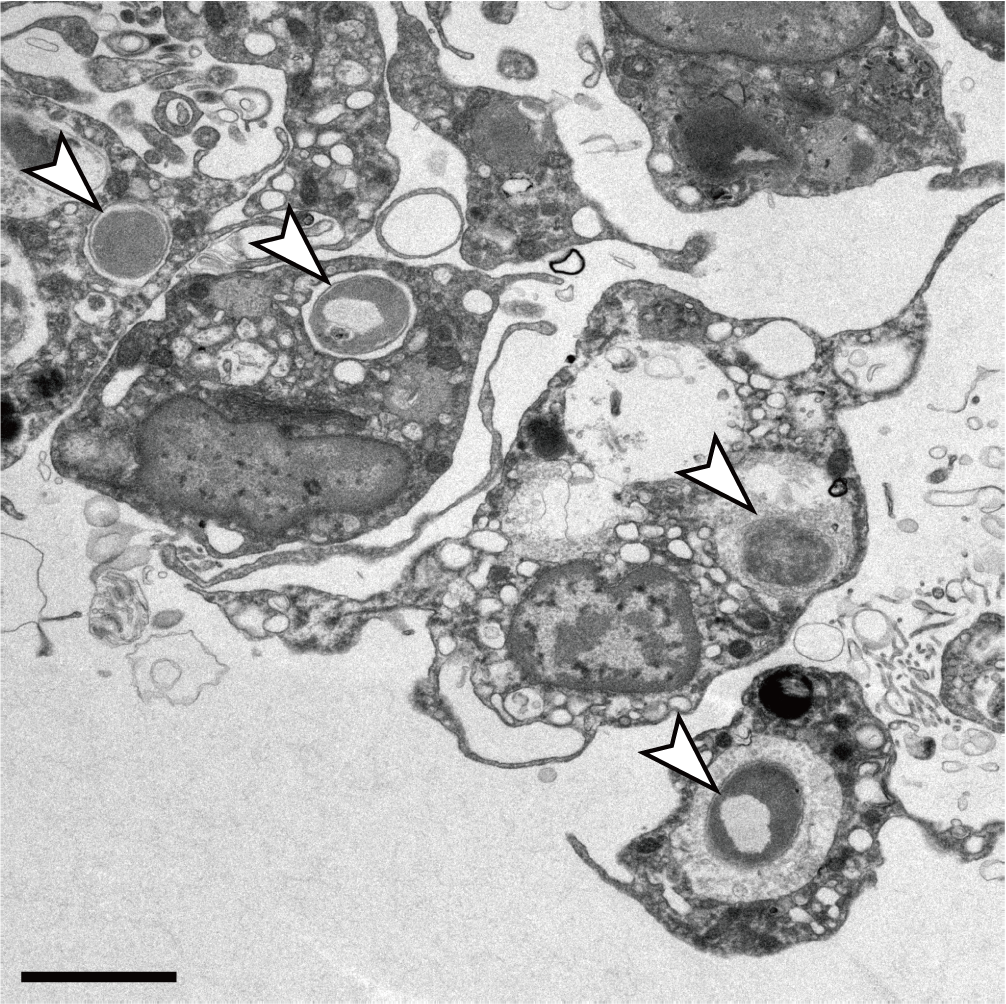

Supplement: Supplementary Figure 3 — Coelomocyte that formed an aggregate in response to injected Escherichia coli. Magnified image of outer edge of the aggregate. Bacterial challenge was performed according to a previously described method [Furukawa et al., Dev Comp Immunol. (2012) 36, 51–61]. Arrowheads indicate phagocytosed bacteria. Similar to the allorecognition response, the interspace of each coelomocyte that phagocytosed the bacteria was filled with pseudopodia, indicating that the coelomocytes did not fuse with each other but phagocytosed the bacteria individually. Scale bar: 2 µm. [file Image_3.tif]

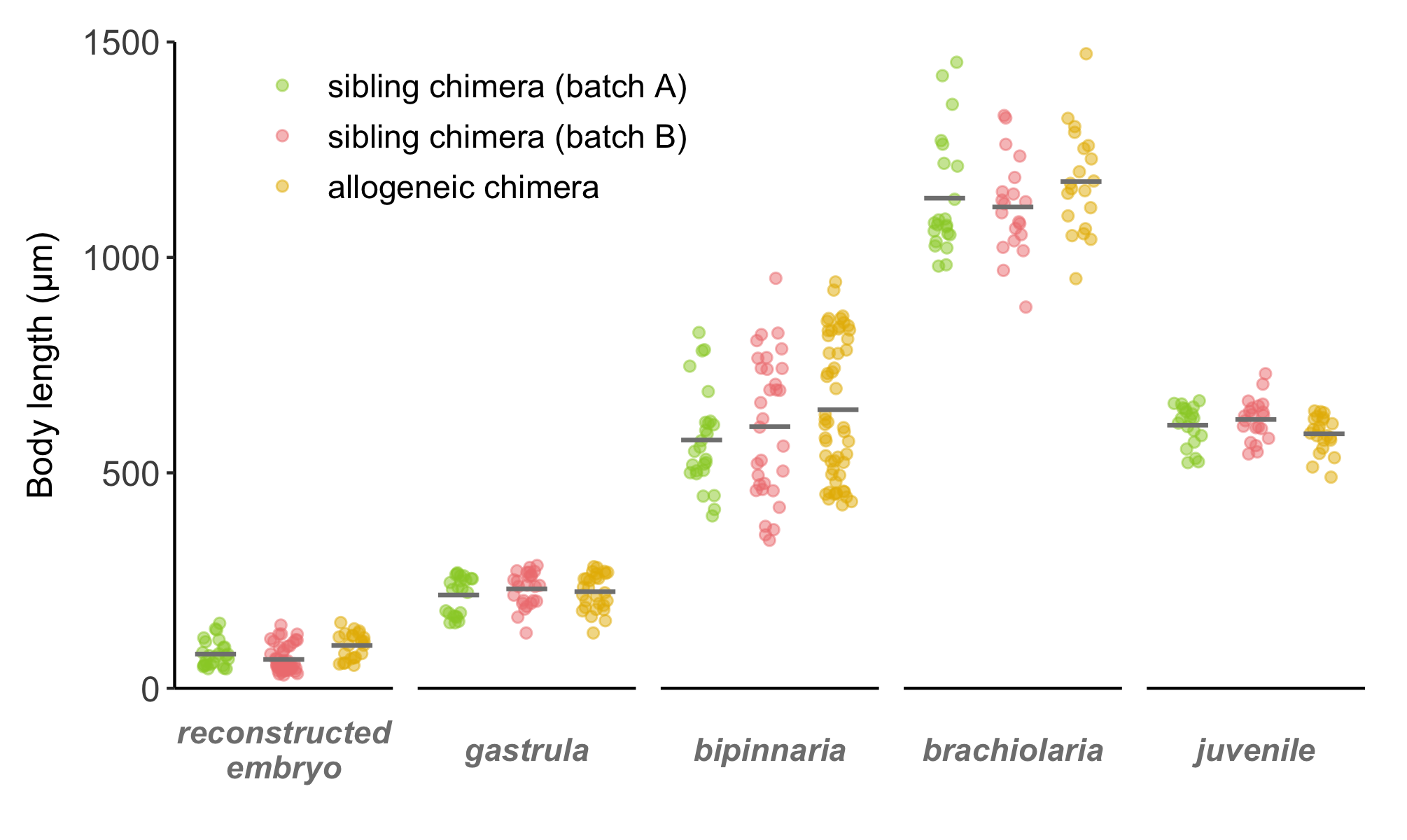

Supplement: Supplementary Figure 4 — Body length of chimeric individuals in each developmental stage. Body length (long diameter) of reconstructed chimera was measured and plotted. No significant differences were detected in each developmental stage according to analysis of variance (ANOVA) tests. [file Image_4.tif]
